# Comparative electrocardiographic study of the Asian freshwater box turtle *Cuora flavomarginata* and the Asian yellow pond turtle *Mauremys mutica* using non‐invasive methods

**DOI:** 10.1002/vro2.52

**Published:** 2022-12-08

**Authors:** Chin‐Chia Kuo, I‐Ping Chan, Cheng‐Hung Lai

**Affiliations:** ^1^ Department of Veterinary Medicine College of Veterinary Medicine National Chung Hsing University Taichung Taiwan; ^2^ Chung Jen Animal Hospital Taichung Taiwan; ^3^ Veterinary Medical Teaching Hospital, National Chung Hsing University Taichung Taiwan

## Abstract

**Background:**

Evaluation of cardiac function is an integral part of clinical examination of chelonians. However, information about electrocardiography (ECG) in turtles and tortoises is limited and fragmentary. Its application is limited due to the lack of ECG reference values. This study aimed to compare specific ECG parameters using non‐invasive methods in the Asian box turtle *Cuora flavomarginata* (CF) and the Asian yellow pond turtle *Mauremys mutica* (MM).

**Methods:**

We included 116 clinically healthy and conscious turtles. Two non‐invasive methods, using adhesive patches or crocodile clips, for ECG were applied where possible. The ambient temperature was within the preferred optimum temperature zone of both species. We used specific digital ECG monitoring equipment to record the ECG data and analysed the data using specific software.

**Results:**

The MM group showed better ECG quality and lower heart rate than the CF group. Comparing both methods, the adhesive patches method yielded higher ECG quality in the CF group, while the crocodile clips method yielded higher ECG quality in the MM group.

**Conclusions:**

The study population was selected as presumed healthy turtles; the presence of systemic or cardiac disease could not be excluded completely due to limited investigation. Both ECG methods were clinically potentially useful for obtaining ECG parameters; the ECG quality was influenced by the method used.

## INTRODUCTION

*Cuora flavomarginata* (CF) and *Mauremys mutica* (MM) are indigenous freshwater turtles in Taiwan.^1^ They belong to the Geomydidae family and live in low‐altitude and high‐humidity forests. Therefore, the climate in Taiwan is appropriate for their growth. They are categorised as endangered on the IUCN Red List; due to illegal overhunting and trade, their population has declined rapidly in recent decades.^2^ Therefore, effective conservation management should be instituted promptly. Establishing baseline values and measurements of the activity and health parameters of these animals is useful for improving the health of the population as the part of their conservation management.^3^

Clinical examination is used to evaluate the health status of turtles and tortoises. Their unique body structure and uncooperative attitude make these examinations difficult for veterinarians. Poor attention has been paid to the evaluation of cardiac performance and diagnosis of heart disease in turtles. The main difficulty in assessing the electrocardiograms of turtles is due to the presence of the shell. It is believed that the electrical signals might be blocked by hard tissues, such as carapace, plastron and bones.^4^ The chelonian heart structure is different from that of mammals. The turtle heart is a three‐chambered structure composed of two atria and a single ventricle, partially divided by a muscular ridge.^5^ Heart muscle contraction in turtles is also different from that in mammals and birds where electrical activation starts from sinoatrial node (SA node) and then sequentially propagates to the atrioventricular node (AV node), bundle of His and Purkinje fibre system. The significant difference between mammals and birds is the angle of mean electrical axis (MEA).^6^ There is no SA node, AV node, bundle of His or Purkinje fibre network that form the specialised conduction system in turtles and tortoises.^7^ The electrical impulse originates from the sinus vein (SV wave), followed by atrial contraction (P wave) and ventricular contraction (QRS complex). The SV waves are often not recognisable in electrocardiography (ECG) owing to their small voltage. After ventricular contraction, the T wave indicates the repolarisation of the ventricle.

The resting metabolic rate of turtles is greatly influenced by the ambient temperature and their specific physiological status. The resting metabolic rate depends on adequate tissue perfusion.^8^ Hence, cardiac output is closely related to the metabolic rate.^5^, ^7^ The heart and metabolic rates increase and decrease when the environmental temperature increases and decreases, respectively.^9^

Electrocardiography is a useful diagnostic tool for specific cardiac diseases. Due to the scarcity of ECG parameter references and standard recording techniques, ECG is rarely performed in turtles and tortoises. Each turtle species has a different body structure and ECG performance. Several studies have reported the performance of ECG in specific chelonian species with invasive and non‐invasive methods.^10^, ^11^, ^12^ Invasive methods have included use of anaesthesia drugs and insertion of probes into the body, which presents the potential risk of harming the animals’ health. None of these studies obtained an ideal ECG recording quality and they were also limited by small sample sizes. Therefore, developing non‐invasive ECG measurement methods are important for evaluating and maintaining the health of chelonians subjects.

The aim of this study was to compare specific parameters using non‐invasive methods in CF and MM. In addition, we explored the factors that influence the interpretation of specific ECG parameters.

## MATERIALS AND METHODS

### Animals and study design

We collected all the turtles that had been confiscated by a rescue facility in a semi‐outdoor enclosure with natural exposure to outdoor temperature, photoperiods and other environmental factors, located in Taichung, Taiwan. The husbandry environment contained one pond (1.6 × 1.6 × 1.2 m for the MM group and 2 × 2 × 0.5 m for the CF group) and one sandpit (1.6 × 1.6 × 1.2 m for the MM group and 3 × 5 × 1 m for the CF group). They were fed fresh vegetables, commercial pellets and sometimes with fresh fruit and dried mealworms.

### Clinical assessment

**FIGURE 1:**
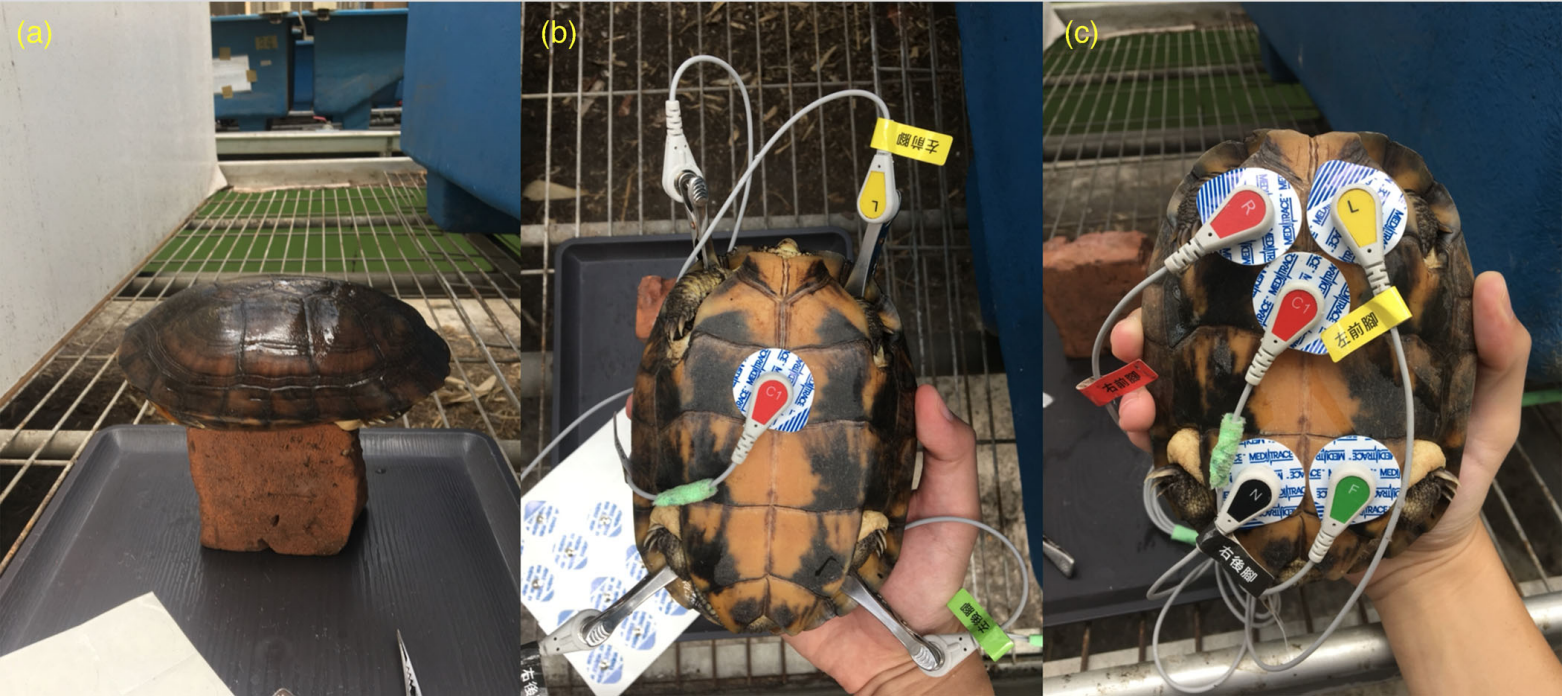
Illustrating the methods used on a turtle to record an electrocardiogram. (a) Turtles were placed on a brick as a restraint device to aid in weighing and examination. (b) Electrocardiography was performed using crocodile clips. A single adhesive patch was applied to the middle of the pectoral scute for one chest lead; crocodile clips were applied to the marginal scute near the extremities for the six limb leads. (c) Electrocardiography was performed using adhesive patches, which were applied to the humeral scute and femoral scute of the plastron for the six limb leads and the middle of pectoral scute for one chest lead.

All turtles were sampled between September 2019 and March 2020. We prospectively included 116 clinically healthy turtles of both species (69 CF and 47 MM) in this study. None of them was under sedation or anaesthesia. We sampled each turtle and measured the bodyweight (BW), maximum straight carapace length (SCL) and maximum curve carapace length (CCL). Simultaneously, we measured the ambient temperature and humidity using an electronic thermo‐hygrometer (Dr. AV, Taiwan). To record the ECG, we placed each turtle on a brick (Figure 1a) as a restraint device for weighing and examining the turtle. The turtles were conscious and resting in the dorsal–ventral position during examination to avoid stress affecting the heart rate (HR).

We used two non‐invasive ECG methods. In the first method, we applied a single adhesive patch (Covidien, Ireland) to the middle of the pectoral scute for one chest lead and crocodile clips to the marginal scute near the extremities for six limb leads (Figure 1b). In the second method, we applied adhesive patches to the humeral scute and femoral scute of the plastron (Figure 1c) for six limb leads and the middle of the pectoral scute for one chest lead. Both ECG methods were applied whenever possible. The ambient temperature was within room temperature and was the preferred optimum temperature zone (POTZ) of both species during ECG measurement (POTZ of CF: 22–30°C; POTZ of MM: 20–30°C). Each ECG sample collection lasted for over 3 min. Sex was determined by examining gonads; all turtles with SCL smaller than 100 mm were classified as juveniles.

### Device and ECG measurement

**FIGURE 2:**
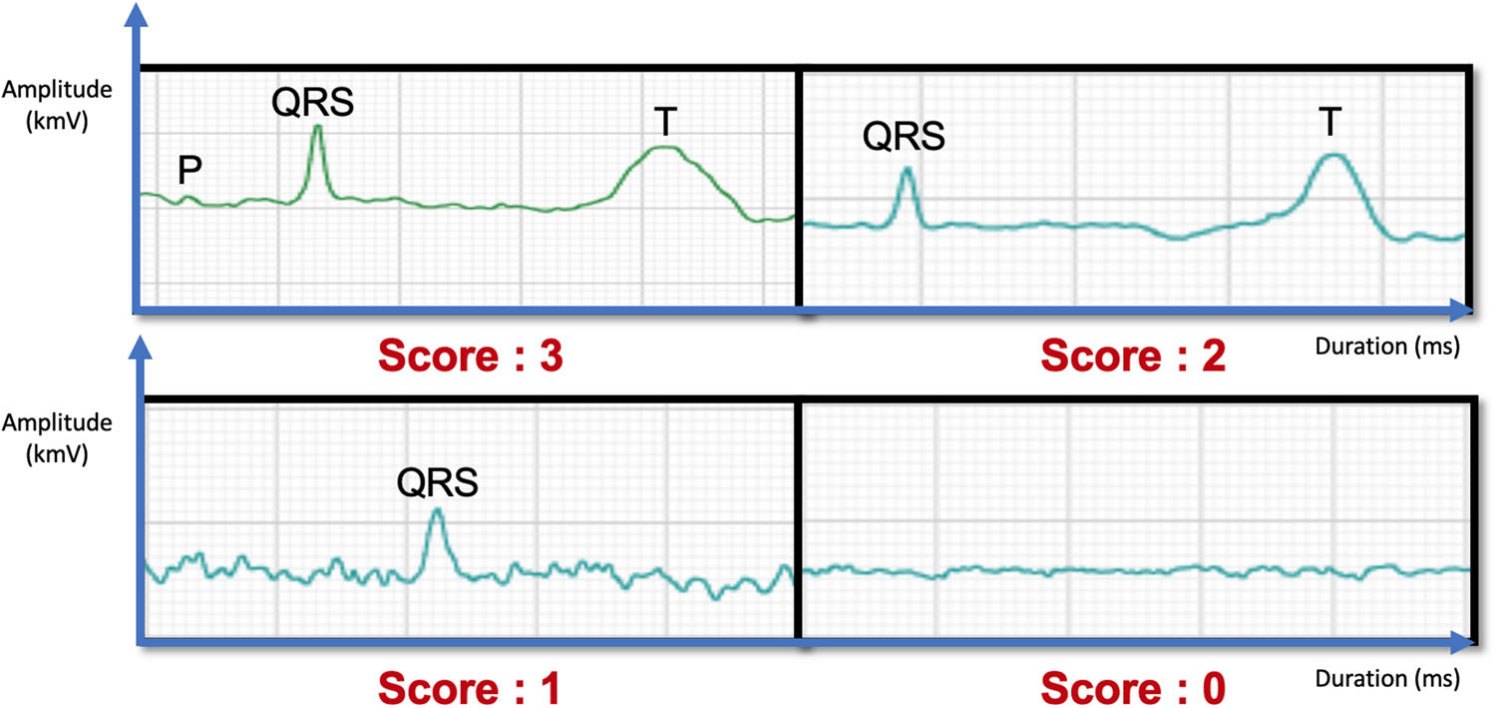
Illustrations of the electrocardiogram (ECG) recording quality—evaluated based on the numbers of recognisable P wave, QRS complex and T wave in lead II. The ECG quality score was graded from 0 to 3, wherein 3 = all of P wave, QRS complex and T wave were recognisable in lead II; 2 = two of P wave, QRS complex and T wave were recognisable in lead II; 1 = only one of P wave, QRS complex and T wave was recognisable in lead II; 0 = none of P wave, QRS complex or T wave was recognisable in lead II

We used mobile, digital and Bluetooth ECG monitoring equipment (Beecardia, Israel) to record ECG samples in seven leads: lead I, lead II, lead III, lead aVR, lead aVL, lead aVF and lead Vx. All collected data were analysed and measured using a website‐based software program (www.beecardia.com). All ECG samples were standardised for voltage at 40 mm = 1 mV and a paper speed of 25 or 50 mm/s. We measured the duration and amplitude of the P wave, QRS complex, T wave, PR interval, ST segment and QT interval. Each ECG variable was measured from five average recognisable heartbeats in each lead. We calculated the HR (beats per minute, bpm) from the number of QRS complexes in a randomly selected and recognisable 30‐s ECG recording. We evaluated the quality of the ECG recording based on the number of recognisable waves of the P wave, QRS complex and T wave in lead II. We graded the ECG quality score from 0 to 3, in which 3 = all of the P wave, QRS complex and T wave could be recognised in lead II; 2 = two of the P wave, QRS complex and T wave could be recognised in lead II; 1 = only one of the P wave, QRS complex and T wave could be recognised in lead II; 0 = none of the P wave, QRS complex and T wave could be recognised in lead II. A higher ECG quality score indicated a better ECG recording quality (Figure 2).

### Statistical analysis

Statistical analyses were performed using the Statistical Package for the Social Sciences software version 25.0 (IBM). For continuous data, we tested normality using the Kolmogorov–Smirnov test. Continuous data were presented as mean ± SD. Comparisons between species, sex and ECG variables were assessed using an independent *t*‐test. The comparison between different ECG methods and specific ECG variables was assessed using a paired *t*‐test. The relationship between clinical characteristics and specific ECG variables was assessed using a two‐tailed Pearson correlation. Statistical significance was set at *p* < 0.05.

## RESULTS

### Animal population

We prospectively enrolled 87 CF and 54 MM in the study between September 2019 and March 2020. We excluded five animals in the CF group and two animals in the MM group due to apparent limb impairment (CF group, *n* = 3; MM group, *n* = 1) and cracked shell (CF group, *n* = 2; MM group, *n* = 1). Four CF and five MM were excluded because of incomplete characteristic records. The final study population consisted of 69 CF and 47 MM, which met the inclusion criteria and were included in the analysis.

### Clinical characteristics

In the CF group, there were 22 males and 47 females. The mean BW, mean SCL and mean CCL were 637.88 ± 275.24 g (range: 177–974 g), 156.12 ± 27.41 mm (range: 103–190 mm) and 180.15 ± 29.74 mm (range: 118–212 mm), respectively. The ambient temperature and humidity during ECG were 25.86 ± 3.10°C (range: 22.5°C–28.7°C) and 59.91 ± 5.91% (range: 46%–69%), respectively.

In the MM group, there were 20 males, 21 females and six juveniles. The mean BW, mean SCL and mean CCL were 400.45 ± 229.84 g (range: 101–1315 g), 137.52 ± 32.05 mm (range: 90–190 mm) and 150.64 ± 33.27 mm (range: 99–242 mm), respectively. The ambient temperature and humidity during ECG were 24.98 ± 1.78°C (range: 22.5°C–28.9°C) and 58.00 ± 7.02% (range: 48%–69%), respectively. The clinical variables of the turtles are summarised in Table 1.

**TABLE 1:** Summary of the clinical variables of the turtles enrolled in the study

Clinical variable	*Cuora flavomarginata* group (*N* = 69)	*Mauremys mutica* group (*N* = 47)
Bodyweight (g)	637.88 ± 275.24	400.45 ± 229.84
Maximum straight carapace length (mm)	156.12 ± 27.4	137.52 ± 32.05
Maximum curve carapace length (mm)	180.15 ± 29.74	150.64 ± 33.27
Sex		
Male	22 (31.9%)	20 (42.6%)
Female	47 (68.1%)	21 (44.7%)
Juvenile	0 (0%)	6 (12.8%)
Ambient temperature (°C)	25.86 ± 3.10	24.98 ± 1.78
Humidity (%)	59.91 ± 5.91	58.00 ± 7.02

### Electrocardiography parameters

#### The CF group

The ECG method using adhesive patches was available in 57 of 69 turtles (82.6%); while using crocodile clips was available in 28 of 69 turtles (40.6%). In both the methods, the largest QRS complex amplitude was revealed in lead II (adhesive patch group, 33/57; crocodile clip group, 16/28). The proportions of the largest QRS complex amplitudes in different leads are summarised in Figure S1.

In the ECGs obtained using the adhesive patch method in lead II, P waves were recognised in 16 turtles; 15 (93.4%) turtles had positive P waves and one (6.6%) had biphasic P waves. R waves were recognised in 57 turtles, all of which were positive. T waves were recognised in 17 turtles; seven (41.2%) turtles had positive T waves and 10 (58.8%) had negative T waves. The ECG quality score was 1.32 ± 0.98.

In the ECGs obtained using the crocodile clips method in lead II, P waves were recognised in eight turtles; seven (87.5%) had positive P waves and one (12.5%) had biphasic P waves. R waves were recognised in 28 turtles, all of which were positive. T waves were recognised in 10 turtles; six (60.0%) had positive T waves and four (40.0%) had negative T waves. The ECG quality score was 0.67 ± 0.95. The ECG parameters in lead II of the CF group are summarised in Tables 2 and 3.

**TABLE 2:** Summary of the electrocardiography parameters in lead II for *Cuora flavomarginata* (CF) and *Mauremys mutica* (MM) groups

Lead II	CF group (clips)	CF group (patch)	MM group (clips)	MM group (patch)
P wave duration (ms)	64.28 ± 18.11 (*N* = 8)	72.88 ± 17.41 (*N* = 16)	75.14 ± 17.32 (*N* = 7)	77.67 ± 7.77 (*N* = 3)
P wave amplitude (kmV)	31.69 ± 16.59 (*N* = 8)	32.94 ± 16.20 (*N* = 16)	30.60 ± 12.60 (*N* = 7)	14.60 ± 11.26 (*N* = 3)
R wave duration (ms)	100.13 ± 24.91 (*N* = 28)	95.58 ± 27.12 (*N* = 57)	89.36 ± 18.31 (*N* = 44)	89.31 ± 16.20 (*N* = 42)
R wave amplitude (kmV)	205.08 ± 118.51 (*N* = 28)	188.53 ± 96.35 (*N* = 57)	185.59 ± 78.54 (*N* = 44)	197.55 ± 77.87 (*N* = 42)
T wave duration (ms)	148.58 ± 46.30 (*N* = 10)	166.06 ± 72.68 (*N* = 18)	299.60 ± 88.81 (*N* = 40)	267.77 ± 79.78 (*N* = 34)
T wave amplitude (kmV)	50.82 ± 162.39 (*N* = 10)	–38.21 ± 106.00 (*N* = 18)	156.31 ± 87.33 (*N* = 40)	158.34 ± 84.27 (*N* = 34)
PR interval (ms)	323.51 ± 159.80 (*N* = 8)	336.13 ± 100.66 (*N* = 16)	342.86 ± 66.42 (*N* = 7)	436.00 ± 38.16 (*N* = 3)
QT interval (ms)	882.58 ± 277.72 (*N* = 10)	844.22 ± 240.51 (*N* = 18)	1117.93 ± 258.29 (*N* = 40)	1008.59 ± 218.04 (*N* = 34)
ST segment (ms)	622.08 ± 245.56 (*N* = 10)	577.50 ± 212.33 (*N* = 18)	727.88 ± 217.84 (*N* = 40)	648.91 ± 203.33 (*N* = 34)

Abbreviation: ms, milliseconds

**TABLE 3:** Summary of the electrocardiography (ECG) quality score for the *Cuora flavomarginata* (CF) and *Mauremys mutica* (MM) groups

Lead II	CF group (clips)	CF group (patch)	MM group (clips)	MM group (patch)
Mean ECG quality score	0.67 ± 0.95	1.32 ± 0.98	1.94 ± 0.70	1.68 ± 0.76
ECG quality score = 0	*N* = 41	*N* = 12	*N* = 3	*N* = 5
ECG quality score = 1	*N* = 15	*N* = 36	*N* = 4	*N* = 8
ECG quality score = 2	*N* = 8	*N* = 13	*N* = 33	*N* = 31
ECG quality score = 3	*N* = 5	*N* = 8	*N* = 7	*N* = 3
Total population	*N* = 69	*N* = 69	*N* = 47	*N* = 47

#### The MM group

The ECG method using adhesive patches was applied in 42 of 47 turtles (89.4%); while using crocodile clips was applied in 44 of 47 turtles (93.6%). For both the methods, the largest QRS complex amplitude was revealed in lead II (adhesive patch group, 28/42; crocodile clip group, 35/44). The proportions of the largest QRS complex amplitudes in different leads are summarised in Figure S1.

In the ECGs obtained using the adhesive patch method in lead II, P waves were recognised in three turtles and were positive. R waves were recognised in 42 turtles, all of which had positive R waves. T waves were recognised in 34 turtles, all of which were positive. The ECG quality score was 1.68 ± 0.76.

In the ECGs obtained using the crocodile clips method in lead II, P waves were recognised in seven turtles and were positive. R waves were recognised in 44 turtles, all of which were positive. T waves were recognised in 40 turtles and were all positive. The ECG quality score was 1.94 ± 0.70. The ECG parameters in lead II of the MM group are summarised in Tables 2 and 3.

#### Species differences

The CF group had a significantly higher HR (mean HR with patch method: CF 35.30 bpm, MM 23.54 bpm; mean HR with clips method: CF 32.48 bpm, MM 20.34 bpm) and lower ECG quality score than the MM group (mean ECG quality score with patch method: CF 1.32, MM 1.68; mean ECG quality score with clips method: CF 0.67, MM 1.94). There were no significant differences between the mean QRS complex amplitude between the CF and MM groups for both ECG methods. The differences between the two species are summarised in Table 4 and Figure S2.

**TABLE 4:** The differences in heart rate (HR), electrocardiography (ECG) quality score and mean QRS complex wave amplitude between the *Cuora flavomarginata* (CF) and *Mauremys mutica* (MM) groups

Variables	CF group	MM group	*p*‐Value
Heart rate (beats per minute, bpm) (patch)	35.28 ± 14.69 (*N* = 57)	23.36 ± 11.82 (*N* = 42)	**<0.001**
Heart rate (bpm) (clips)	31.89 ± 12.80 (*N* = 28)	20.61 ± 10.83 (*N* = 44)	**<0.001**
Electrocardiography quality score (patch)	1.32 ± 0.98 (*N* = 69)	1.68 ± 0.76 (*N* = 47)	**0.027**
Electrocardiography quality score (clips)	0.67 ± 0.95 (*N* = 69)	1.94 ± 0.70 (*N* = 47)	**<0.001**
R wave amplitude in lead II (kmV) (patch)	188.53 ± 96.35 (*N* = 56)	197.55 ± 77.87 (*N* = 42)	0.620
R wave amplitude in lead II (kmV) (clips)	205.08 ± 118.51 (*N* = 28)	185.59 ± 78.54 (*N* = 44)	0.446

*Note*: There were significant differences in heart rate (patch), heart rate (clips), ECG quality score (patch) and ECG quality score (clips) between the CF and MM groups.

Bold values means the significant differences between the two different groups.

#### Sex difference

In the CF and MM groups, there were no significant differences in HR, ECG quality score or mean QRS complex amplitude between male and female turtles.

#### Method differences

In the CF group, the ECGs obtained using adhesive patches showed significantly higher ECG quality scores than those using crocodile clips. There was no significant difference in HR or mean QRS complex amplitudes between the two ECG methods.

In the MM group, the ECGs obtained using adhesive patches showed significantly lower ECG quality scores than those using crocodile clips. There was no significant difference in HR or mean QRS complex amplitudes between the two ECG methods. The group differences between the different ECG methods are summarised in Table 5 and Figure S2.

**TABLE 5:** The differences in heart rate (HR), electrocardiography (ECG) quality score and mean QRS complex wave amplitude between different ECG methods in the *Cuora flavomarginata* (CF) and *Mauremys mutica* (MM) groups

Variables	Patch method	Clips method	*p*‐Value
CF group			
Heart rate (beats per minute, bpm)	35.30 ± 13.06 (*N* = 27)	32.48 ± 12.65 (*N* = 27)	0.091
ECG quality score	1.32 ± 0.98 (*N* = 69)	0.67 ± 0.95 (*N* = 69)	**<0.001**
R wave amplitude in lead II (kmV)	208.57 ± 107.60 (*N* = 26)	211.14 ± 120.96 (*N* = 26)	0.851
MM group			
Heart rate (bpm)	23.54 ± 11.91 (*N* = 41)	20.34 ± 10.09 (*N* = 41)	0.144
Electrocardiography quality score	1.68 ± 0.76 (*N* = 47)	1.94 ± 0.70 (*N* = 47)	**0.017**
R wave amplitude in lead II (kmV)	198.74 ± 78.46 (*N* = 41)	187.66 ± 76.41 (*N* = 41)	0.102

*Note*: There were significant differences in the ECG quality score between different ECG methods in the CF and MM groups.

Bold values means the significant differences between the two different groups.

### Correlation analysis

In both groups, ECGs obtained using either adhesive patches or crocodile clips showed that the correlations between BW, CCL, SCL, ambient temperature versus HR, ECG quality score and mean QRS complex amplitude were not statistically significant under the conditions of the study.

## DISCUSSION

To the best of the authors’ knowledge, this is the first study to assess ECGs using different ECG methods and the correlation between clinical characteristics and specific ECG parameters in CF and MM. Both ECG methods used in this study were clinically useful for ECG recording. The present study also showed specific ECG parameters for both species in lead II, including the duration and amplitude of the P wave, QRS complex, T wave, PR interval, ST segment, QT interval and HR.

Several reports have described ECG methods used for turtles and tortoises. A previous study performed ECG in a red‐eared slider (*Trachemys scripta elegans*) under anaesthesia with the use of non‐invasive alligator clips. They connected the electrodes to the skin overlying the right forelimb, left forelimb and left hindlimb.^11^ A previous study performed ECG in 20 different species of conscious turtles with two different ECG methods. Fixation of the ECG electrodes was performed using adhesive patches or clamp electrodes attached to the extremities, skin folds or plaston.^12^ In our study, we used clips and applied electrodes to the skin folds of the limbs of both species. Most of the turtles waved their limbs to jerk away from the clips, especially in the CF group. This technique may not be easy to apply in conscious turtles. Similar to a previous report,^12^ we used adhesive patches and crocodile clips as ECG electrodes. When electrodes are attached to the limbs, turtles often struggle to get free of the electrodes. The ECG quality was better with adhesive patches than with crocodile clips because of turtle struggling. When attaching electrodes to the carapace, the ECG waveforms were too small to be recognised using adhesive patches; however, the ECG waveforms were clear and could be recognised using crocodile clips. These were the reasons why we decided to apply adhesive patches to the plastron or attach crocodile clips to the carapace for ECG recording. A comparison of different studies of ECG in turtles is summarised in Table S1.^11^, ^12^, ^20^

The characteristics of the ECG waveforms in this study resemble those of other studies.^11^, ^20^ Similar to those reports, the deflection of the P wave and QRS complex in lead II were all positive in CF and MM. Furthermore, T wave deflection in lead II was positive in red‐eared sliders (*Trachemys scripta elegans*) and other species of turtles.^11^, ^20^ In our study, T wave deflection in lead II was positive in the MM group, while it was biphasic in the CF group. The reason why T wave deflection in lead II was biphasic in CF is unclear. Compared to mammals, CF and MM showed very long QT intervals. These results are similar to those of other species of reptiles. In addition, SV waves were not recognisable in CF and MM because of the small voltage in the ECG recordings. This result is similar to that reported by other authors.^1^, ^2^, ^11^, ^12^, ^20^

Electrocardiography is a useful diagnostic tool for heart diseases. This technique has been used and reported in many different ‘exotic’ species.^12^, ^13^, ^14^, ^15^, ^16^, ^17^, ^18^, ^19^ Due to the scant information on ECG references, it is rarely performed in turtles and tortoises. The MEA is the average of all the instantaneous mean electrical vectors that occur sequentially during depolarisation of the ventricles. Each animal species had a reference MEA range. A shift in MEA may indicate specific ventricular enlargement or conduction disturbances. To calculate the angle of the MEA in the frontal plane, we measured the amplitude of the QRS complex in leads I and III or in leads I and aVF, then added the vector of both leads to obtain the resultant magnitude and direction. In mammals, birds and some reptiles, such as snakes, the QRS complex in leads I, III and aVF are clearly recognisable.^6^, ^21^ Hence, it can be used to calculate the MEA. However, in the present study, the QRS complex amplitude in lead I was very small or buried in the baseline, which did not allow those signals to be interpreted in most of the turtles. Due to this result, we could not accurately measure the MEA of the QRS complex in CF and MM. We noticed that a high proportion of the population in both species had similar ECG waveforms and the electrical axis was deflected in lead II. Hence, we speculated that turtles with abnormal ECG waveforms or MEA instead of lead II might have cardiac disease. Further investigation including diagnostic imaging examination could be used to clarify whether structural cardiac disease is present. No diagnostic imaging examination was performed in the current study.

We observed significant variation between the two species in the present study. We found that the MM group had a lower HR and better quality ECG than those of the CF group using the same ECG method. Comparing both species, CF was more hyperactive and sensitive to this approach than MM. *Cuora flavomarginata* is a species of the Asian box turtle. They are characterised by a domed shell, hinged at the bottom, allowing the animal to retract its head and legs and close its shell tightly. Therefore, the response when performing ECG was often waving their limbs or closing their shells tightly. This response led to difficulty in ECG assessment and produced artefacts in the ECG recording, causing poor ECG quality in this group. In addition, their motor activity and irritability also led to an increased HR. In contrast to the CF group, the MM group was usually stunned or had no significant response during the approach; therefore, better ECG quality and lower HR were obtained.

In both species, sex was not a significant influencing factor for HR, ECG quality or mean QRS complex amplitude. In a previous study, ECG in five different species of turtles reported that the female QRS complex amplitude was higher than that of the males.^10^ Differences in populations and clinical conditions among studies may lead to differences in results. Due to the different species and small sample size of each species in other studies, the authors could not draw conclusions regarding the correlation between sex and specific ECG variables.

Both ECG methods produced results depending on the species. In the present study, HR and mean QRS complex amplitude were not significantly different between the two ECG methods. Better ECG quality was obtained using adhesive patches in the CF group and crocodile clips in the MM group. Due to the large interspecies variability in turtles and tortoises, the authors did not expect that a single ECG method could be used in both species of turtle. Compared to applying adhesive patches to shells, fixation of the electrodes to the skin folds of the carapace resulted in fewer artefacts. Theoretically, this method offers better ECG quality for recognising specific ECG waveform recordings. However, the CF group was more hyperactive and resisted the approach, making fixation of the electrodes more difficult using crocodile clips; therefore, using adhesive patches was more suitable for this species. In contrast, the MM group was calmer, making both ECG methods easy to perform; therefore, using crocodile clips was better because of fewer artefacts.

Turtles are poikilothermic animals, so ambient temperature is an important factor when assessing them.^7^ There is a close relationship between HR and environmental temperature in turtles and tortoises.^22^ Many species of reptiles have a faster HR during warming and a slower HR during cooling.^5^, ^23^ In our study, ambient temperature was not considered an influencing factor for HR. All turtles were examined within the POTZ; therefore, these turtles had good metabolic functioning during the assessment. Thermally induced changes in HR and blood flow in reptiles are thought to allow these animals to regulate metabolism. In turtles and tortoises, HR is closely correlated with ambient temperature.^22^ Within a similar ambient temperature, there may be no significant difference in HR between turtles.

In addition, BW and body size were not significantly correlated with HR, ECG quality or mean QRS complex amplitude in CF and MM. In mammals, the HR is usually negatively correlated with BW.^24^ Although few reports have described the relationship between BW and body size—or HR and QRS complex amplitude in turtles—BW and body size were not important factors for evaluating HR in either species.

This study had several limitations. First, the study population was relatively small for both species. This limitation might have reduced the statistical power. However, the sample size was larger than that in previous studies. Second, there were twice as many females as males in the CF group, which could have introduced a sex bias that was not identifiable on some ECG parameters. Third, the study population was selected as apparently healthy turtles; the presence of systemic or cardiac disease may not be excluded completely from the study population due to limited investigation through clinical examination and additionally routine blood examination and echocardiography. *Cuora flavomarginata* and MM are listed as endangered species in Taiwan. Any invasive method (such as blood sampling) should be applied and approved by the specific competent authority before assessment. Fourth, these turtles were kept in the shelter at specific environmental temperatures and humidity. The study population may not represent wild turtles of the same species. Fifth, the influence of the plastron, such as different bony components, was not evaluated. Due to legal limitations, more invasive methods, such as drilling the plastron to collect bony sample in alive protected animals were prohibited. Finally, all ECG recordings were obtained with the animals in the ventral side position. Therefore, the ECG parameters obtained in our study may not be applicable to ECG recordings in other positions.

## AUTHOR CONTRIBUTIONS

Chin‐Chia Kuo and I‐Ping Chan conceived the idea of the study. Chin‐Chia Kuo conceived and designed the study and was involved in acquisition, analysis and interpretation of data. All authors contributed to the revision of the article and to the writing in its current form and approved the final version.

## CONFLICTS OF INTEREST

The authors declare they have no conflicts of interest.

## FUNDING INFORMATION

This study was self‐funded.

## ETHICS STATEMENT

This study was approved by the Institutional Animal Care and Use Committee (approval number: 108‐089) and protected animal applications (number: 109‐育利019).

## Supporting information

Supporting Information

## Data Availability

There is no data statement.
