# Supplementary material for: Comparative electrocardiographic study of the Asian freshwater box turtle Cuora flavomarginata and the Asian yellow pond turtle Mauremys mutica using non‐invasive methods
Source: Vet Rec Open. 2022 Dec 8;9(1):e52. doi: 10.1002/vro2.52 (PMC9732382; doi:10.1002/vro2.52)
Supplement: Supplementary file 1 — Supporting Information [file VRO2-9-e52-s001.docx]

# Supporting Information

# Table S1 A comparison of different studies of electrocardiography in turtles

| **Reference** | **Number of species** | **Number of animals examined** | **Method** | **Anaesthesia (Y/N)** | **Invasive (Y/N)** | **Attached place** | **Results** |
| --- | --- | --- | --- | --- | --- | --- | --- |
| 10 | 1 | 9 | Clip | Y | Y | Skin of limbs | Detected ECG waves in nine animals. |
| 19 | unknown | 37 | Clip | N | N | Skin of limbs | No P waves detected in 14 animals.  No T waves detected in 10 animals. |
| 11 | 20 | 72 | Patch  Clip | N | N | Plastron  Skin of limbs | Detected ECG waves in 25 animals (patch).  Detected ECG waves in 11 animals. (clip) |
| The current study | 2 | 116 | Patch  Clip | N | N | Plastron  Skin of carapace | Detected ECG waves in 99 animals (patch).  Detected ECG waves in 72 animals. (clip) |

# Figure S1 Number of turtles with the largest QRS complex amplitude in different leads for the Asian pond turtles ***Cuora flavomarginata* (CF) and *Mauremys mutica* (MM)**


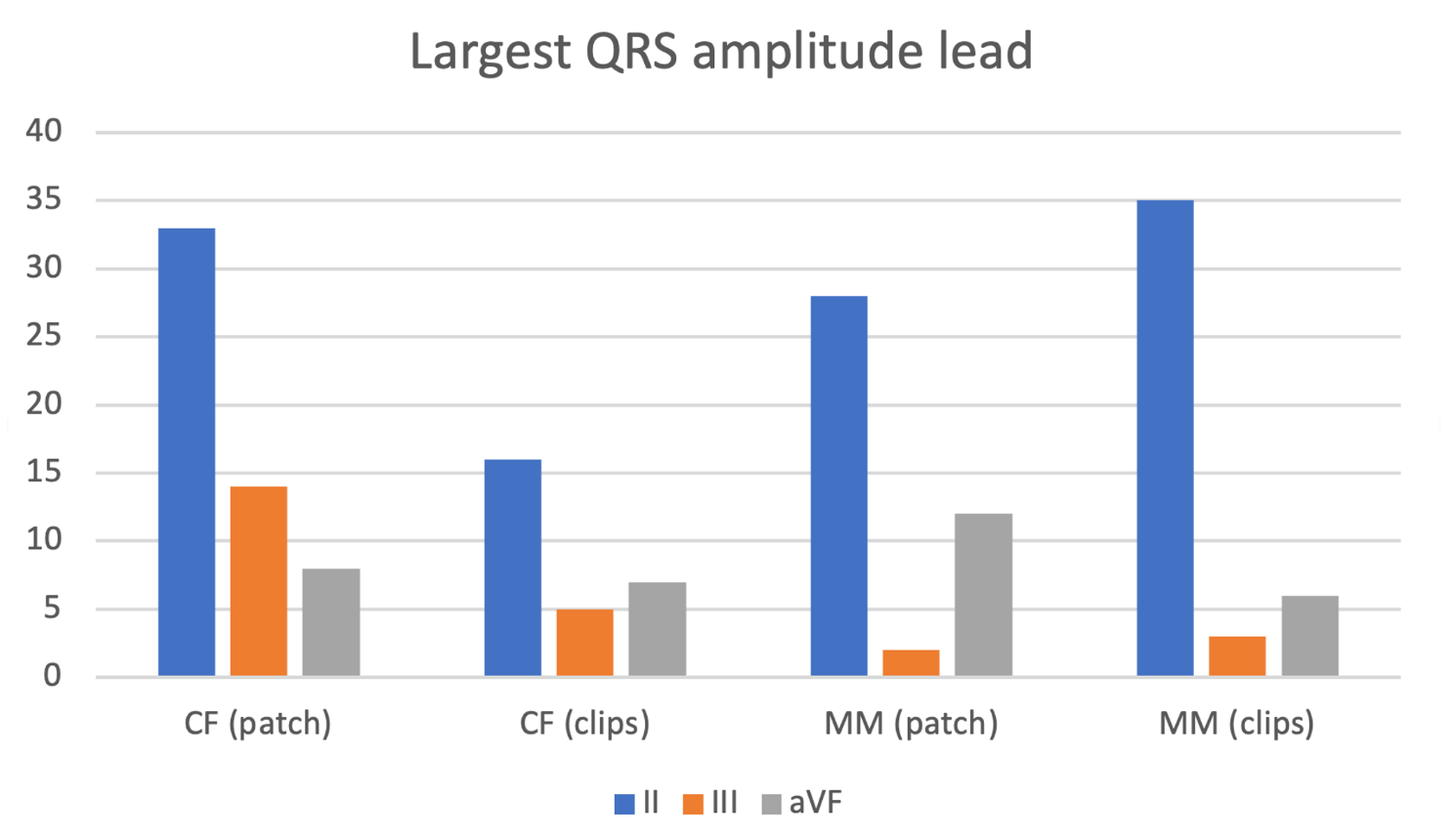


Regardless of the methods and species examined, the largest QRS complex amplitude was most commonly revealed in lead II.

# Figure S2 Differences in heart rate and electrocardiography (ECG) quality score between the pond turtles ***Cuora flavomarginata* (CF) and *Mauremys mutica* (MM) and between two** ECG methods


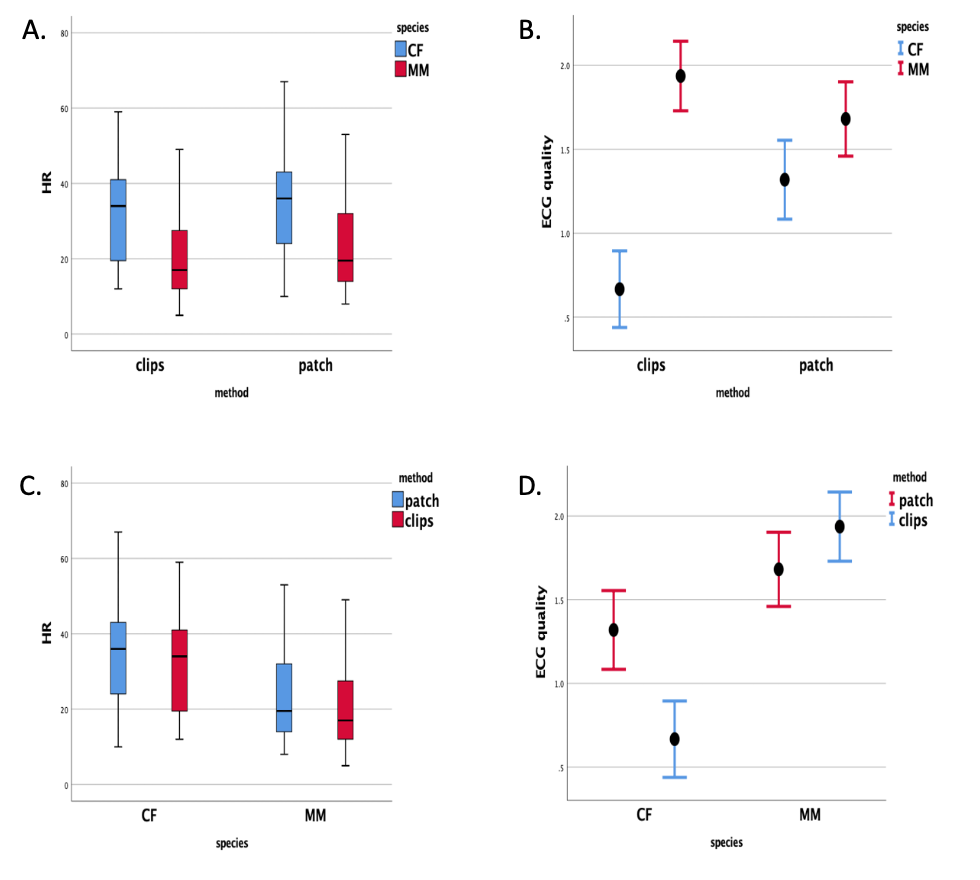


(A, B) In the group allocated by species, performing ECG using adhesive patches or crocodile clips, the *Cuora flavomarginata* group had significantly higher HR and lower ECG quality score than those of the *Mauremys mutica* group with P value < 0.05.

(C, D) In the group allocated by ECG methods, there was no significant difference in heart rate between different ECG methods in both species.

In the *Cuora flavomarginata* group, a higher ECG quality score was obtained using adhesive patches. While in the *Mauremys mutica* group, a higher ECG quality score was obtained using crocodile clips.
